# Supplementary material for: Immune Evasion of SARS-CoV-2 Omicron Subvariants XBB.1.5, XBB.1.16 and EG.5.1 in a Cohort of Older Adults after ChAdOx1-S Vaccination and BA.4/5 Bivalent Booster
Source: Vaccines (Basel). 2024 Jan 30;12(2):144. doi: 10.3390/vaccines12020144 (PMC10892985; doi:10.3390/vaccines12020144)
Supplement: Supplementary file 1 [file vaccines-12-00144-s001.zip › vaccines-2742186-supplementary.pdf]

Supplemental Table S1. Summary of patient's information (n=59). Identification (ID), race, day of birth (DOB), age (years), sex (F, female or M, male), comorbidities, previously SARS-CoV-2 reported infection (RT-qPCR positivity, date and Ct value), epidemiological Omicron wave based on date of infection (see materials and methods for details) and days post infection (DPI) and 1st blood collection on November 10, 2022. Ct, cycle threshold. N/A, not applicable.

Participant ID	Race	DOB	Age (years)	Sex (F/M)	Comorbidities	RT-qPCR positive for SARS-CoV-2	Ct value	Date of RT-qPCR positivity for SARS-CoV-2	Epidemiological Omicron wave	Days post infection and 1st blood collection (11/10/22)
318775	Mixed	17/09/1947	75	M	Dysmobility syndrome	Positive	19	1/24/22	BA.1/BA.2	290
421648	White	03/06/1937	85	F	Alzheimer's disease	Positive	36	7/8/22	BA.4/BA.5	125
323845	White	21/07/1952	70	F	Dementia	Positive	32	1/21/22	BA.1/BA.2	293
379131	White	11/11/1953	69	F	Dementia	Positive	22	7/8/22	BA.4/BA.5	125
267871	White	12/07/1947	75	F	Dysmobility syndrome	Positive	24	1/18/22	BA.1/BA.2	296
267367	Black	12/10/1958	64	M	Rheumatic fever	Positive	23	6/13/22	BA.4/BA.5	150
230397	White	02/09/1959	63	F	Schizophrenia	Negative	N/A	N/A	N/A	N/A
494837	Asian	08/06/1938	84	F	Hypertension	Negative	N/A	N/A	N/A	N/A
394951	Mixed	21/08/1944	78	F	Dementia	Positive	17	6/21/22	BA.4/BA.5	142
346858	White	14/07/1950	72	M	Dysmobility syndrome	Negative	N/A	N/A	N/A	N/A
406477	Mixed	11/12/1948	74	M	Dementia	Positive	22	1/24/22	BA.1/BA.2	290
267519	Black	31/12/1953	68	M	Hypertension	Positive	20	1/26/22	BA.1/BA.2	288
407102	Mixed	19/06/1960	62	M	None	Negative	N/A	N/A	N/A	N/A
298184	White	17/06/1956	66	M	Hypertension	Positive	25	6/20/22	BA.4/BA.5	143
378877	White	13/06/1925	97	M	Dysmobility syndrome	Positive	22	6/20/22	BA.4/BA.5	143
304913	Mixed	19/10/1956	66	M	Dysmobility syndrome	Negative	N/A	N/A	N/A	N/A
267864	Black	15/03/1951	71	M	Hypertension	Positive	23	6/13/22	BA.4/BA.5	150
267549	Mixed	19/10/1956	66	M	Sequeale of stroke	Negative	N/A	N/A	N/A	N/A
476825	Mixed	17/03/1957	65	M	Dysmobility syndrome	Negative	N/A	N/A	N/A	N/A
476761	White	11/07/1941	81	F	Hypertension	Positive	37	7/1/22	BA.4/BA.5	132
476919	Asian	16/05/1949	73	M	None	Negative	N/A	N/A	N/A	N/A
267420	Black	05/02/1934	88	F	Sequale of Traumatic Brain Injury (TBI)	Positive	23	6/30/22	BA.4/BA.5	133
355937	White	08/03/1938	84	F	None	Negative	N/A	N/A	N/A	N/A
359150	White	30/09/1946	76	F	Hypertension	Positive	30	1/18/22	BA.1/BA.2	296
112553	White	21/03/1950	72	F	Dysmobility syndrome	Positive	22	6/20/22	BA.4/BA.5	143
111333	White	19/08/1947	75	F	Dysmobility syndrome and cerebral palsy	Positive	29	1/18/22	BA.1/BA.2	296
271671	Black	04/08/1957	65	F	Sequeale of stroke	Negative	N/A	N/A	N/A	N/A
198987	Mixed	02/11/1932	90	F	Dementia	Negative	N/A	N/A	N/A	N/A
323515	Black	07/03/1936	86	F	Schizophrenia	Positive	22	7/12/22	BA.4/BA.5	121
320888	White	19/02/1948	74	F	Dementia	Positive	18	4/25/22	BA.1/BA.2	199
267765	Black	21/01/1937	85	F	Dementia	Positive	22	1/18/22	BA.1/BA.2	296
110443	White	17/11/1942	80	F	Schizophrenia	Positive	28	1/14/22	BA.1/BA.2	300
401522	Mixed	29/09/1949	73	F	Dementia	Positive	21	6/21/22	BA.4/BA.5	142
380501	White	23/12/1940	81	F	Type 2 diabetes mellitus (T2DM)	Positive	19	1/28/22	BA.1/BA.2	286
176972	Black	04/07/1941	81	F	Sequeale of stroke	Negative	N/A	N/A	N/A	N/A
336759	White	14/07/1939	83	F	Schizophrenia	Negative	N/A	N/A	N/A	N/A
482490	Mixed	13/12/1953	69	M	Dementia	Negative	N/A	N/A	N/A	N/A
477571	Mixed	28/11/1960	62	M	Type 2 diabetes mellitus (T2DM) and hypertension	Positive	22	1/26/22	BA.1/BA.2	288
192130	Black	20/10/1955	67	F	Hypertension	Positive	18	8/7/22	BA.4/BA.5	125
985	Black	19/11/1957	65	F	Dysmobility syndrome	Positive	24	2/2/22	BA.1/BA.2	281
440135	White	18/10/1957	65	M	Dementia	Negative	N/A	N/A	N/A	N/A
442378	White	13/01/1958	64	M	Leprosy	Positive	31	6/21/22	BA.4/BA.5	142
440447	Mixed	01/03/1957	65	M	Dementia	Positive	32	6/22/22	BA.4/BA.5	141
450144	White	19/05/1943	79	F	Dementia	Negative	N/A	N/A	N/A	N/A
447167	Mixed	21/11/1939	83	M	Sequeale of stroke	Positive	29	6/22/22	BA.4/BA.5	141
267652	Black	29/06/1957	65	M	Sequeale of stroke	Positive	23	6/21/22	BA.4/BA.5	142
471697	White	25/01/1950	72	M	Dysmobility syndrome	Negative	N/A	N/A	N/A	N/A
199379	Black	28/08/1945	77	M	Sequeale of stroke	Positive	22	1/24/22	BA.1/BA.2	290
370201	Black	28/10/1941	81	M	Dysmobility syndrome	Positive	25	1/24/22	BA.1/BA.2	290
267502	Black	28/06/1952	70	M	Sequale of Traumatic Brain Injury (TBI)	Positive	19	1/24/22	BA.1/BA.2	290
333440	White	08/05/1944	78	M	Dysmobility syndrome	Positive	16	1/24/22	BA.1/BA.2	290
267520	Mixed	03/06/1954	68	M	Sequeale of stroke	Negative	N/A	N/A	N/A	N/A
28511	Black	10/01/1942	80	M	Dysmobility syndrome	Negative	N/A	N/A	N/A	N/A
402327	Mixed	08/03/1944	78	M	Dementia	Positive	22	3/23/21	BA.1/BA.2	597
363357	White	20/04/1949	73	M	Sequeale of stroke	Positive	20	1/28/22	BA.1/BA.2	286
290602	Mixed	11/02/1954	68	M	Dysmobility syndrome	Negative	N/A	N/A	N/A	N/A
160442	Mixed	12/08/1952	70	M	Hypertension	Positive	22	1/24/22	BA.1/BA.2	290
211607	White	08/10/1938	84	M	None	Negative	N/A	N/A	N/A	N/A
466183	White	31/05/1943	79	M	Dementia	Negative	N/A	N/A	N/A	N/A

Supplemental Table S2. Vaccination records from all the participants of the study (n=49). Identification (ID), the full vaccination record against COVID-19, including which vaccine, the date of administration and the vaccine batch number.

ID	Vaccine 1st dose	Vaccine batch number	1st dose date	Vaccine 2nd dose	Vaccine batch number	2nd dose date	Vaccine 3rd dose	Vaccine batch number	3rd dose date	Vaccine 4th dose	Vaccine batch number	4th dose date	Vaccine 5th dose	Vaccine batch number	5th dose date
318775	ChAdOx1-S (Oxford/AstraZeneca)	4120Z005	5-Feb-21	ChAdOx1-S (Oxford/AstraZeneca)	213VCD020W	11-May-21	BNT162b2 (Pfizer-BioNTech)	FJ4187	30-Nov-21	ChAdOx1-S (Oxford/AstraZeneca)	221VCD006Z	7-Oct-22	Comirnaty Bivalent Original/Omicron BA.4/BA.5 (Pfizer-BioNTech)	GJ2556	2-Mar-23
421648	ChAdOx1-S (Oxford/AstraZeneca)	4120Z005	5-Feb-21	ChAdOx1-S (Oxford/AstraZeneca)	213VCD020W	11-May-21	BNT162b2 (Pfizer-BioNTech)	FJ4187	30-Nov-21	ChAdOx1-S (Oxford/AstraZeneca)	221VCD006Z	5-Oct-22	Comirnaty Bivalent Original/Omicron BA.4/BA.5 (Pfizer-BioNTech)	GJ2556	2-Mar-23
323845	ChAdOx1-S (Oxford/AstraZeneca)	4120Z005	5-Feb-21	ChAdOx1-S (Oxford/AstraZeneca)	213VCD020W	12-May-21	BNT162b2 (Pfizer-BioNTech)	FJ4187	30-Nov-21	ChAdOx1-S (Oxford/AstraZeneca)	221VCD006Z	5-Oct-22	Comirnaty Bivalent Original/Omicron BA.4/BA.5 (Pfizer-BioNTech)	GJ2556	2-Mar-23
379131	ChAdOx1-S (Oxford/AstraZeneca)	4120Z005	5-Feb-21	ChAdOx1-S (Oxford/AstraZeneca)	213VCD020W	29-Apr-21	BNT162b2 (Pfizer-BioNTech)	FJ4187	30-Nov-21	ChAdOx1-S (Oxford/AstraZeneca)	221VCD006Z	5-Oct-22	Comirnaty Bivalent Original/Omicron BA.4/BA.5 (Pfizer-BioNTech)	GJ2556	2-Mar-23
267871	ChAdOx1-S (Oxford/AstraZeneca)	4120Z005	5-Feb-21	ChAdOx1-S (Oxford/AstraZeneca)	213VCD020W	11-May-21	BNT162b2 (Pfizer-BioNTech)	FJ4187	30-Nov-21	ChAdOx1-S (Oxford/AstraZeneca)	221VCD006Z	6-Oct-22	Comirnaty Bivalent Original/Omicron BA.4/BA.5 (Pfizer-BioNTech)	GJ2556	2-Mar-23
267367	ChAdOx1-S (Oxford/AstraZeneca)	4120Z005	5-Feb-21	ChAdOx1-S (Oxford/AstraZeneca)	213VCD020W	11-May-21	BNT162b2 (Pfizer-BioNTech)	FJ4187	30-Nov-21	ChAdOx1-S (Oxford/AstraZeneca)	221VCD006Z	7-Oct-22	Comirnaty Bivalent Original/Omicron BA.4/BA.5 (Pfizer-BioNTech)	GJ2556	3-Mar-23
230397	ChAdOx1-S (Oxford/AstraZeneca)	4120Z005	5-May-21	ChAdOx1-S (Oxford/AstraZeneca)	213VCD020W	30-Jul-21	BNT162b2 (Pfizer-BioNTech)	FJ4187	28-Jan-22	ChAdOx1-S (Oxford/AstraZeneca)	221VCD006Z	5-Oct-22	Comirnaty Bivalent Original/Omicron BA.4/BA.5 (Pfizer-BioNTech)	GJ2556	2-Mar-23
494837	ChAdOx1-S (Oxford/AstraZeneca)	41207026	1-Mar-21	ChAdOx1-S (Oxford/AstraZeneca)	214VCD061Z	24-May-21	BNT162b2 (Pfizer-BioNTech)	FJ4187	2-Dec-21	ChAdOx1-S (Oxford/AstraZeneca)	221VCD006Z	7-Oct-22	Comirnaty Bivalent Original/Omicron BA.4/BA.5 (Pfizer-BioNTech)	GJ2556	3-Mar-23
394951	ChAdOx1-S (Oxford/AstraZeneca)	4120Z005	5-Feb-21	ChAdOx1-S (Oxford/AstraZeneca)	213VCD020W	11-May-21	BNT162b2 (Pfizer-BioNTech)	FJ4187	30-Nov-21	ChAdOx1-S (Oxford/AstraZeneca)	221VCD006Z	6-Oct-22	Comirnaty Bivalent Original/Omicron BA.4/BA.5 (Pfizer-BioNTech)	GJ2556	2-Mar-23
346858	ChAdOx1-S (Oxford/AstraZeneca)	4120Z005	5-Feb-21	ChAdOx1-S (Oxford/AstraZeneca)	213VCD020W	11-May-21	BNT162b2 (Pfizer-BioNTech)	FJ4187	30-Nov-21	ChAdOx1-S (Oxford/AstraZeneca)	221VCD006Z	7-Oct-22	Comirnaty Bivalent Original/Omicron BA.4/BA.5 (Pfizer-BioNTech)	GJ2556	3-Mar-23
406477	ChAdOx1-S (Oxford/AstraZeneca)	4120Z005	5-Feb-21	ChAdOx1-S (Oxford/AstraZeneca)	213VCD020W	11-May-21	BNT162b2 (Pfizer-BioNTech)	FJ4187	30-Nov-21	ChAdOx1-S (Oxford/AstraZeneca)	221VCD006Z	6-Oct-22	Comirnaty Bivalent Original/Omicron BA.4/BA.5 (Pfizer-BioNTech)	GJ2556	2-Mar-23
267519	ChAdOx1-S (Oxford/AstraZeneca)	4120Z005	5-Feb-21	ChAdOx1-S (Oxford/AstraZeneca)	213VCD020W	11-May-21	BNT162b2 (Pfizer-BioNTech)	FJ4187	30-Nov-21	ChAdOx1-S (Oxford/AstraZeneca)	221VCD006Z	7-Oct-22	Comirnaty Bivalent Original/Omicron BA.4/BA.5 (Pfizer-BioNTech)	GJ2556	3-Mar-23
407102	ChAdOx1-S (Oxford/AstraZeneca)	4120Z005	5-Feb-21	ChAdOx1-S (Oxford/AstraZeneca)	213VCD020W	29-Apr-21	BNT162b2 (Pfizer-BioNTech)	FJ4187	30-Nov-21	ChAdOx1-S (Oxford/AstraZeneca)	221VCD006Z	5-Oct-22	Comirnaty Bivalent Original/Omicron BA.4/BA.5 (Pfizer-BioNTech)	GJ2556	2-Mar-23
298184	ChAdOx1-S (Oxford/AstraZeneca)	4120Z005	5-Feb-21	ChAdOx1-S (Oxford/AstraZeneca)	213VCD020W	11-May-21	BNT162b2 (Pfizer-BioNTech)	FJ4187	30-Nov-21	ChAdOx1-S (Oxford/AstraZeneca)	221VCD006Z	5-Oct-22	Comirnaty Bivalent Original/Omicron BA.4/BA.5 (Pfizer-BioNTech)	GJ2556	2-Mar-23
378877	ChAdOx1-S (Oxford/AstraZeneca)	4120Z005	5-Feb-21	ChAdOx1-S (Oxford/AstraZeneca)	213VCD020W	11-May-21	BNT162b2 (Pfizer-BioNTech)	FJ4187	30-Nov-21	ChAdOx1-S (Oxford/AstraZeneca)	221VCD006Z	5-Oct-22	Comirnaty Bivalent Original/Omicron BA.4/BA.5 (Pfizer-BioNTech)	GJ2556	2-Mar-23
304913	ChAdOx1-S (Oxford/AstraZeneca)	4120Z005	5-Feb-21	ChAdOx1-S (Oxford/AstraZeneca)	213VCD020W	11-May-21	BNT162b2 (Pfizer-BioNTech)	FJ4187	30-Nov-21	ChAdOx1-S (Oxford/AstraZeneca)	221VCD006Z	5-Oct-22	Comirnaty Bivalent Original/Omicron BA.4/BA.5 (Pfizer-BioNTech)	GJ2556	2-Mar-23
267864	ChAdOx1-S (Oxford/AstraZeneca)	4120Z005	5-Feb-21	ChAdOx1-S (Oxford/AstraZeneca)	213VCD020W	11-May-21	BNT162b2 (Pfizer-BioNTech)	FJ4187	30-Nov-21	ChAdOx1-S (Oxford/AstraZeneca)	221VCD006Z	7-Oct-22	Comirnaty Bivalent Original/Omicron BA.4/BA.5 (Pfizer-BioNTech)	GJ2556	3-Mar-23
267549	ChAdOx1-S (Oxford/AstraZeneca)	4120Z005	5-Feb-21	ChAdOx1-S (Oxford/AstraZeneca)	213VCD020W	11-May-21	BNT162b2 (Pfizer-BioNTech)	FJ4187	30-Nov-21	ChAdOx1-S (Oxford/AstraZeneca)	221VCD006Z	6-Oct-22	Comirnaty Bivalent Original/Omicron BA.4/BA.5 (Pfizer-BioNTech)	GJ2556	3-Mar-23
476825	ChAdOx1-S (Oxford/AstraZeneca)	4120Z005	5-Feb-21	ChAdOx1-S (Oxford/AstraZeneca)	213VCD020W	29-Apr-21	BNT162b2 (Pfizer-BioNTech)	FJ4187	30-Nov-21	ChAdOx1-S (Oxford/AstraZeneca)	221VCD006Z	4-Oct-22	Comirnaty Bivalent Original/Omicron BA.4/BA.5 (Pfizer-BioNTech)	GJ2556	10-Mar-23
476761	ChAdOx1-S (Oxford/AstraZeneca)	4120Z005	5-Feb-21	ChAdOx1-S (Oxford/AstraZeneca)	213VCD020W	29-Apr-21	BNT162b2 (Pfizer-BioNTech)	FJ4187	30-Nov-21	ChAdOx1-S (Oxford/AstraZeneca)	221VCD006Z	5-Oct-22	Comirnaty Bivalent Original/Omicron BA.4/BA.5 (Pfizer-BioNTech)	GJ2556	2-Mar-23
476919	ChAdOx1-S (Oxford/AstraZeneca)	4120Z005	5-Feb-21	ChAdOx1-S (Oxford/AstraZeneca)	213VCD020W	11-May-21	BNT162b2 (Pfizer-BioNTech)	FJ4187	30-Nov-21	ChAdOx1-S (Oxford/AstraZeneca)	221VCD006Z	7-Oct-22	Comirnaty Bivalent Original/Omicron BA.4/BA.5 (Pfizer-BioNTech)	GJ2556	3-Mar-23
267420	ChAdOx1-S (Oxford/AstraZeneca)	4120Z005	5-Feb-21	ChAdOx1-S (Oxford/AstraZeneca)	213VCD020W	11-May-21	BNT162b2 (Pfizer-BioNTech)	FJ4187	30-Nov-21	ChAdOx1-S (Oxford/AstraZeneca)	221VCD006Z	5-Oct-22	Comirnaty Bivalent Original/Omicron BA.4/BA.5 (Pfizer-BioNTech)	GJ2556	2-Mar-23
355937	ChAdOx1-S (Oxford/AstraZeneca)	4120Z005	5-Feb-21	ChAdOx1-S (Oxford/AstraZeneca)	213VCD020W	11-May-21	BNT162b2 (Pfizer-BioNTech)	FJ4187	30-Nov-21	ChAdOx1-S (Oxford/AstraZeneca)	221VCD006Z	5-Oct-22	Comirnaty Bivalent Original/Omicron BA.4/BA.5 (Pfizer-BioNTech)	GJ2556	2-Mar-23
359150	ChAdOx1-S (Oxford/AstraZeneca)	4120Z005	5-Feb-21	ChAdOx1-S (Oxford/AstraZeneca)	213VCD020W	11-May-21	BNT162b2 (Pfizer-BioNTech)	FJ4187	30-Nov-21	ChAdOx1-S (Oxford/AstraZeneca)	221VCD006Z	5-Oct-22	Comirnaty Bivalent Original/Omicron BA.4/BA.5 (Pfizer-BioNTech)	GJ2556	2-Mar-23
112553	ChAdOx1-S (Oxford/AstraZeneca)	4120Z005	5-Feb-21	ChAdOx1-S (Oxford/AstraZeneca)	213VCD020W	11-May-21	BNT162b2 (Pfizer-BioNTech)	FJ4187	30-Nov-21	ChAdOx1-S (Oxford/AstraZeneca)	221VCD006Z	5-Oct-22	Comirnaty Bivalent Original/Omicron BA.4/BA.5 (Pfizer-BioNTech)	GJ2556	2-Mar-23
111333	ChAdOx1-S (Oxford/AstraZeneca)	4120Z005	5-Feb-21	ChAdOx1-S (Oxford/AstraZeneca)	213VCD020W	11-May-21	BNT162b2 (Pfizer-BioNTech)	FJ4187	30-Nov-21	ChAdOx1-S (Oxford/AstraZeneca)	221VCD006Z	5-Oct-22	Comirnaty Bivalent Original/Omicron BA.4/BA.5 (Pfizer-BioNTech)	GJ2556	2-Mar-23
271671	ChAdOx1-S (Oxford/AstraZeneca)	4120Z005	5-Feb-21	ChAdOx1-S (Oxford/AstraZeneca)	213VCD020W	11-May-21	BNT162b2 (Pfizer-BioNTech)	FJ4187	30-Nov-21	ChAdOx1-S (Oxford/AstraZeneca)	221VCD006Z	5-Oct-22	Comirnaty Bivalent Original/Omicron BA.4/BA.5 (Pfizer-BioNTech)	GJ2556	2-Mar-23
198987	ChAdOx1-S (Oxford/AstraZeneca)	4120Z005	5-Feb-21	ChAdOx1-S (Oxford/AstraZeneca)	213VCD020W	11-May-21	BNT162b2 (Pfizer-BioNTech)	FJ4187	30-Nov-21	ChAdOx1-S (Oxford/AstraZeneca)	221VCD006Z	5-Oct-22	Comirnaty Bivalent Original/Omicron BA.4/BA.5 (Pfizer-BioNTech)	GJ2556	2-Mar-23
323515	ChAdOx1-S (Oxford/AstraZeneca)	4120Z005	5-Feb-21	ChAdOx1-S (Oxford/AstraZeneca)	213VCD020W	11-May-21	BNT162b2 (Pfizer-BioNTech)	FJ4187	30-Nov-21	ChAdOx1-S (Oxford/AstraZeneca)	221VCD006Z	5-Oct-22	Comirnaty Bivalent Original/Omicron BA.4/BA.5 (Pfizer-BioNTech)	GJ2556	2-Mar-23
320888	ChAdOx1-S (Oxford/AstraZeneca)	4120Z005	5-Feb-21	ChAdOx1-S (Oxford/AstraZeneca)	213VCD020W	11-May-21	BNT162b2 (Pfizer-BioNTech)	FJ4187	30-Nov-21	ChAdOx1-S (Oxford/AstraZeneca)	221VCD006Z	6-Oct-22	Comirnaty Bivalent Original/Omicron BA.4/BA.5 (Pfizer-BioNTech)	GJ2556	2-Mar-23
267765	ChAdOx1-S (Oxford/AstraZeneca)	4120Z005	5-Feb-21	ChAdOx1-S (Oxford/AstraZeneca)	213VCD020W	11-May-21	BNT162b2 (Pfizer-BioNTech)	FJ4187	30-Nov-21	ChAdOx1-S (Oxford/AstraZeneca)	221VCD006Z	5-Oct-22	Comirnaty Bivalent Original/Omicron BA.4/BA.5 (Pfizer-BioNTech)	GJ2556	2-Mar-23
110443	ChAdOx1-S (Oxford/AstraZeneca)	4120Z005	5-Feb-21	ChAdOx1-S (Oxford/AstraZeneca)	213VCD020W	11-May-21	BNT162b2 (Pfizer-BioNTech)	FJ4187	30-Nov-21	ChAdOx1-S (Oxford/AstraZeneca)	221VCD006Z	6-Oct-22	Comirnaty Bivalent Original/Omicron BA.4/BA.5 (Pfizer-BioNTech)	GJ2556	2-Mar-23
401522	ChAdOx1-S (Oxford/AstraZeneca)	4120Z005	5-Feb-21	ChAdOx1-S (Oxford/AstraZeneca)	213VCD020W	11-May-21	BNT162b2 (Pfizer-BioNTech)	FJ4187	30-Nov-21	ChAdOx1-S (Oxford/AstraZeneca)	221VCD006Z	5-Oct-22	Comirnaty Bivalent Original/Omicron BA.4/BA.5 (Pfizer-BioNTech)	GJ2556	2-Mar-23
380501	ChAdOx1-S (Oxford/AstraZeneca)	4120Z005	5-Feb-21	ChAdOx1-S (Oxford/AstraZeneca)	213VCD020W	11-May-21	BNT162b2 (Pfizer-BioNTech)	FJ4187	30-Nov-21	ChAdOx1-S (Oxford/AstraZeneca)	221VCD006Z	5-Oct-22	Comirnaty Bivalent Original/Omicron BA.4/BA.5 (Pfizer-BioNTech)	GJ2556	3-Mar-23
176972	ChAdOx1-S (Oxford/AstraZeneca)	4120Z005	5-Feb-21	ChAdOx1-S (Oxford/AstraZeneca)	213VCD020W	11-May-21	BNT162b2 (Pfizer-BioNTech)	FJ4187	30-Nov-21	ChAdOx1-S (Oxford/AstraZeneca)	221VCD006Z	6-Oct-22	Comirnaty Bivalent Original/Omicron BA.4/BA.5 (Pfizer-BioNTech)	GJ2556	2-Mar-23
336759	ChAdOx1-S (Oxford/AstraZeneca)	4120Z005	5-Feb-21	ChAdOx1-S (Oxford/AstraZeneca)	213VCD020W	11-May-21	BNT162b2 (Pfizer-BioNTech)	FJ4187	30-Nov-21	ChAdOx1-S (Oxford/AstraZeneca)	221VCD006Z	5-Oct-22	Comirnaty Bivalent Original/Omicron BA.4/BA.5 (Pfizer-BioNTech)	GJ2556	2-Mar-23
482490	ChAdOx1-S (Oxford/AstraZeneca)	4120Z005	5-Feb-21	ChAdOx1-S (Oxford/AstraZeneca)	213VCD020W	11-May-21	BNT162b2 (Pfizer-BioNTech)	FJ4187	30-Nov-21	ChAdOx1-S (Oxford/AstraZeneca)	221VCD006Z	5-Oct-22	Comirnaty Bivalent Original/Omicron BA.4/BA.5 (Pfizer-BioNTech)	GJ2556	2-Mar-23
477571	ChAdOx1-S (Oxford/AstraZeneca)	4120Z005	5-Feb-21	ChAdOx1-S (Oxford/AstraZeneca)	213VCD020W	29-Apr-21	BNT162b2 (Pfizer-BioNTech)	FJ4187	30-Nov-21	ChAdOx1-S (Oxford/AstraZeneca)	221VCD006Z	5-Oct-22	Comirnaty Bivalent Original/Omicron BA.4/BA.5 (Pfizer-BioNTech)	GJ2556	2-Mar-23
192130	ChAdOx1-S (Oxford/AstraZeneca)	4120Z005	5-Feb-21	ChAdOx1-S (Oxford/AstraZeneca)	213VCD020W	12-May-21	BNT162b2 (Pfizer-BioNTech)	FJ4187	30-Nov-21	ChAdOx1-S (Oxford/AstraZeneca)	221VCD006Z	5-Oct-22	Comirnaty Bivalent Original/Omicron BA.4/BA.5 (Pfizer-BioNTech)	GJ2556	2-Mar-23
985	ChAdOx1-S (Oxford/AstraZeneca)	4120Z005	5-Feb-21	ChAdOx1-S (Oxford/AstraZeneca)	213VCD020W	11-May-21	BNT162b2 (Pfizer-BioNTech)	FJ4187	30-Nov-21	ChAdOx1-S (Oxford/AstraZeneca)	221VCD006Z	6-Oct-22	Comirnaty Bivalent Original/Omicron BA.4/BA.5 (Pfizer-BioNTech)	GJ2556	2-Mar-23
440135	ChAdOx1-S (Oxford/AstraZeneca)	4120Z005	5-Feb-21	ChAdOx1-S (Oxford/AstraZeneca)	213VCD020W	11-May-21	BNT162b2 (Pfizer-BioNTech)	FJ4187	30-Nov-21	ChAdOx1-S (Oxford/AstraZeneca)	221VCD006Z	5-Oct-22	Comirnaty Bivalent Original/Omicron BA.4/BA.5 (Pfizer-BioNTech)	GJ2556	5-Mar-23
442378	ChAdOx1-S (Oxford/AstraZeneca)	4120Z005	5-Feb-21	ChAdOx1-S (Oxford/AstraZeneca)	213VCD020W	11-May-21	BNT162b2 (Pfizer-BioNTech)	FJ4187	30-Nov-21	ChAdOx1-S (Oxford/AstraZeneca)	221VCD006Z	5-Oct-22	Comirnaty Bivalent Original/Omicron BA.4/BA.5 (Pfizer-BioNTech)	GJ2556	2-Mar-23
440447	ChAdOx1-S (Oxford/AstraZeneca)	4120Z005	5-Feb-21	ChAdOx1-S (Oxford/AstraZeneca)	213VCD020W	11-May-21	BNT162b2 (Pfizer-BioNTech)	FJ4187	30-Nov-21	ChAdOx1-S (Oxford/AstraZeneca)	221VCD006Z	5-Oct-22	Comirnaty Bivalent Original/Omicron BA.4/BA.5 (Pfizer-BioNTech)	GJ2556	2-Mar-23
450144	ChAdOx1-S (Oxford/AstraZeneca)	4120Z005	5-Feb-21	ChAdOx1-S (Oxford/AstraZeneca)	213VCD020W	28-Apr-21	BNT162b2 (Pfizer-BioNTech)	FJ4187	30-Nov-21	ChAdOx1-S (Oxford/AstraZeneca)	221VCD006Z	5-Oct-22	Comirnaty Bivalent Original/Omicron BA.4/BA.5 (Pfizer-BioNTech)	GJ2556	2-Mar-23
447167	ChAdOx1-S (Oxford/AstraZeneca)	4120Z005	5-Feb-21	ChAdOx1-S (Oxford/AstraZeneca)	213VCD020W	11-May-21	BNT162b2 (Pfizer-BioNTech)	FJ4187	30-Nov-21	ChAdOx1-S (Oxford/AstraZeneca)	221VCD006Z	5-Oct-22	Comirnaty Bivalent Original/Omicron BA.4/BA.5 (Pfizer-BioNTech)	GJ2556	2-Mar-23
267652	ChAdOx1-S (Oxford/AstraZeneca)	4120Z005	5-Feb-21	ChAdOx1-S (Oxford/AstraZeneca)	213VCD020W	11-May-21	BNT162b2 (Pfizer-BioNTech)	FJ4187	30-Nov-21	ChAdOx1-S (Oxford/AstraZeneca)	221VCD006Z	5-Oct-22	Comirnaty Bivalent Original/Omicron BA.4/BA.5 (Pfizer-BioNTech)	GJ2556	7-Mar-23
471697	ChAdOx1-S (Oxford/AstraZeneca)	4120Z005	5-Feb-21	ChAdOx1-S (Oxford/AstraZeneca)	213VCD020W	11-May-21	BNT162b2 (Pfizer-BioNTech)	FJ4187	30-Nov-21	ChAdOx1-S (Oxford/AstraZeneca)	221VCD006Z	5-Oct-22	Comirnaty Bivalent Original/Omicron BA.4/BA.5 (Pfizer-BioNTech)	GJ2556	2-Mar-23
199379	ChAdOx1-S (Oxford/AstraZeneca)	4120Z005	5-Feb-21	ChAdOx1-S (Oxford/AstraZeneca)	213VCD020W	11-May-21	BNT162b2 (Pfizer-BioNTech)	FJ4187	30-Nov-21	ChAdOx1-S (Oxford/AstraZeneca)	221VCD006Z	5-Oct-22	Comirnaty Bivalent Original/Omicron BA.4/BA.5 (Pfizer-BioNTech)	GJ2556	3-Mar-23
370201	ChAdOx1-S (Oxford/AstraZeneca)	4120Z005	5-Feb-21	ChAdOx1-S (Oxford/AstraZeneca)	213VCD020W	11-May-21	BNT162b2 (Pfizer-BioNTech)	FJ4187	30-Nov-21	ChAdOx1-S (Oxford/AstraZeneca)	221VCD006Z	5-Oct-22	Comirnaty Bivalent Original/Omicron BA.4/BA.5 (Pfizer-BioNTech)	GJ2556	2-Mar-23
267502	ChAdOx1-S (Oxford/AstraZeneca)	4120Z005	5-Feb-21	ChAdOx1-S (Oxford/AstraZeneca)	213VCD020W	11-May-21	BNT162b2 (Pfizer-BioNTech)	FJ4187	30-Nov-21	ChAdOx1-S (Oxford/AstraZeneca)	221VCD006Z	5-Oct-22	Comirnaty Bivalent Original/Omicron BA.4/BA.5 (Pfizer-BioNTech)	GJ2556	2-Mar-23
333440	ChAdOx1-S (Oxford/AstraZeneca)	4120Z005	5-Feb-21	ChAdOx1-S (Oxford/AstraZeneca)	213VCD020W	11-May-21	BNT162b2 (Pfizer-BioNTech)	FJ4187	30-Nov-21	ChAdOx1-S (Oxford/AstraZeneca)	221VCD006Z	5-Oct-22	Comirnaty Bivalent Original/Omicron BA.4/BA.5 (Pfizer-BioNTech)	GJ2556	6-Mar-23
267520	ChAdOx1-S (Oxford/AstraZeneca)	4120Z005	5-Feb-21	ChAdOx1-S (Oxford/AstraZeneca)	213VCD020W	11-May-21	BNT162b2 (Pfizer-BioNTech)	FJ4187	30-Nov-21	ChAdOx1-S (Oxford/AstraZeneca)	221VCD006Z	5-Oct-22	Comirnaty Bivalent Original/Omicron BA.4/BA.5 (Pfizer-BioNTech)	GJ2556	2-Mar-23
28511	ChAdOx1-S (Oxford/AstraZeneca)	4120Z005	5-Feb-21	ChAdOx1-S (Oxford/AstraZeneca)	213VCD020W	11-May-21	BNT162b2 (Pfizer-BioNTech)	FJ4187	30-Nov-21	ChAdOx1-S (Oxford/AstraZeneca)	221VCD006Z	6-Oct-22	Comirnaty Bivalent Original/Omicron BA.4/BA.5 (Pfizer-BioNTech)	GJ2556	2-Mar-23
402327	ChAdOx1-S (Oxford/AstraZeneca)	4120Z005	5-Feb-21	ChAdOx1-S (Oxford/AstraZeneca)	213VCD020W	11-May-21	BNT162b2 (Pfizer-BioNTech)	FJ4187	30-Nov-21	ChAdOx1-S (Oxford/AstraZeneca)	221VCD006Z	5-Oct-22	Comirnaty Bivalent Original/Omicron BA.4/BA.5 (Pfizer-BioNTech)	GJ2556	2-Mar-23
363357	ChAdOx1-S (Oxford/AstraZeneca)	4120Z005	5-Feb-21	ChAdOx1-S (Oxford/AstraZeneca)	213VCD020W	11-May-21	BNT162b2 (Pfizer-BioNTech)	FJ4187	30-Nov-21	ChAdOx1-S (Oxford/AstraZeneca)	221VCD006Z	5-Oct-22	Comirnaty Bivalent Original/Omicron BA.4/BA.5 (Pfizer-BioNTech)	GJ2556	2-Mar-23
290602	ChAdOx1-S (Oxford/AstraZeneca)	4120Z005	5-Feb-21	ChAdOx1-S (Oxford/AstraZeneca)	213VCD020W	29-Apr-21	BNT162b2 (Pfizer-BioNTech)	FM3355	17-Dec-21	ChAdOx1-S (Oxford/AstraZeneca)	221VCD006Z	5-Oct-22	Comirnaty Bivalent Original/Omicron BA.4/BA.5 (Pfizer-BioNTech)	GJ2556	2-Mar-23
160442	ChAdOx1-S (Oxford/AstraZeneca)	4120Z005	5-Feb-21	ChAdOx1-S (Oxford/AstraZeneca)	213VCD020W	11-May-21	BNT162b2 (Pfizer-BioNTech)	FJ4187	30-Nov-21	ChAdOx1-S (Oxford/AstraZeneca)	221VCD006Z	5-Oct-22	Comirnaty Bivalent Original/Omicron BA.4/BA.5 (Pfizer-BioNTech)	GJ2556	2-Mar-23
211607	ChAdOx1-S (Oxford/AstraZeneca)	4120Z005	5-Feb-21	ChAdOx1-S (Oxford/AstraZeneca)	213VCD020W	11-May-21	BNT162b2 (Pfizer-BioNTech)	FJ4187	30-Nov-21	ChAdOx1-S (Oxford/AstraZeneca)	221VCD006Z	5-Oct-22	Comirnaty Bivalent Original/Omicron BA.4/BA.5 (Pfizer-BioNTech)	GJ2556	2-Mar-23
466183	ChAdOx1-S (Oxford/AstraZeneca)	4120Z005	5-Feb-21	ChAdOx1-S (Oxford/AstraZeneca)	213VCD020W	11-May-21	BNT162b2 (Pfizer-BioNTech)	FJ4187	30-Nov-21	ChAdOx1-S (Oxford/AstraZeneca)	221VCD006Z	6-Oct-22	Comirnaty Bivalent Original/Omicron BA.4/BA.5 (Pfizer-BioNTech)	GJ2556	2-Mar-23

Supplemental Table S3. Geometric mean titers (GMTs), 95% CI and of neutralizing titers and percentage (%) of seroconversion against SARS-CoV-2 ancestral strain (D614G) and Omicron sublineages BA.4/5, XBB.1.5, XBB.1.16 and EG.5.1 at 1-4 months post 4th dose with ChAdOx1-S (Oxford/AstraZeneca) and 3 months post 5th dose with Comirnaty Bivalent Original/Omicron BA.4/BA.5 (Pfizer-BioNTech). Individual NT_{50} values is the geometric mean (GMT) of triplicate CPE-VNT results (one independent experiment). Titers below the LOD (<20) were represented by half the LOD, 10 for plot purpose and statistical analysis. ID, identification; dpv, days post-vaccination; NT_{50}, 50% neutralization antibody titer; 95% CI, 95% confidence interval.

Participant ID	Neutralization titer (NT50): 1 month (median 36 days) after 4th dose with ChAdOx1-S (Oxford/AstraZeneca)					4 months (median 126 days) after 4th dose with ChAdOx1-S (Oxford/AstraZeneca)					3 months (median 91 days) after Comirnaty Bivalent Original/Omicron BA.4/BA.5 (Pfizer-BioNTech)				
	D614G	BA.4	XBB 1.5	XBB 1.16	EG.5.1	D614G	BA.4	XBB 1.5	XBB 1.16	EG.5.1	D614G	BA.4	XBB 1.5	XBB 1.16	EG.5.1
440135	3620	1810	453	226	113	1810	905	226	22	71	2874	3620	570	453	570
442378	3620	905	22	18	45	2874	905	71	14	57	3620	3620	180	180	113
440447	3620	3620	28	14	22	3620	1437	71	14	28	3620	2874	71	143	143
450144	1140	226	28	18	36	1140	285	22	14	14	3620	3620	718	359	180
447167	3620	905	226	143	113	226	90	14	14	14	2874	905	143	143	143
267652	226	453	180	71	143	453	2281	453	143	143	1437	2874	905	453	570
471697	718	453	36	28	36	2281	1810	113	45	36	2874	3620	570	285	143
199379	1810	2874	905	285	453	570	226	22	14	22	1140	1810	453	143	359
370201	3620	3620	2281	905	905	2874	453	90	36	45	3620	3620	1810	359	905
267502	2281	453	113	45	71	1437	905	180	57	90	2874	3620	2281	2281	905
333440	905	570	359	143	90	3620	71	45	14	36	2874	1437	180	113	71
267520	570	285	113	28	180	2281	905	90	22	36	2874	1140	180	143	143
28511	3620	2874	905	453	57	2281	718	180	57	57	2874	718	180	71	90
402327	3620	1140	226	113	113	2281	905	226	90	57	3620	3620	453	718	453
363357	2874	1437	570	359	180	2874	359	90	45	57	2874	1810	285	226	285
290602	2874	2281	226	71	180	1437	113	22	28	28	3620	1437	453	226	453
160442	113	90	18	18	45	2281	2281	57	18	22	3620	2874	180	143	226
211607	2874	1140	143	57	36	45	90	18	14	14	2874	3620	285	285	285
466183	2281	3620	905	285	90	2874	3620	285	113	113	1810	1810	90	143	113
406477	718	1437	143	57	90	359	90	14	14	14	453	2281	285	226	143
267519	1810	1810	71	57	90	2281	1437	359	71	90	1810	1140	226	226	180
407102	3620	3620	285	57	180	718	36	14	14	14	3620	3620	180	180	71
298184	905	718	45	14	36	1437	718	71	36	45	2874	718	71	90	90
378877	1140	570	113	36	90	570	226	71	18	57	1437	570	113	71	143
304913	1810	453	45	18	14	359	71	14	14	14	2281	905	45	45	36
267864	905	285	71	28	90	905	718	90	36	57	359	570	180	113	285
267549	2281	285	45	22	57	2874	359	180	71	143	1810	226	180	57	143
476825	3620	1140	180	113	285	2281	1140	226	36	71	3620	3620	905	1140	453
476761	2281	3620	2281	1810	905	1140	226	71	36	71	905	3620	2281	1140	905
476919	113	14	14	14	14	285	71	14	14	14	1437	285	28	45	28
267420	1810	2874	359	180	285	2281	2281	143	57	180	2874	3620	905	359	285
355937	1810	570	36	14	14	1140	905	28	14	45	1437	359	71	28	71
359150	3620	1810	453	113	180	3620	905	71	28	28	1810	718	285	143	57
112553	3620	1140	180	143	285	3620	1810	45	36	28	2874	2874	285	180	226
111333	3620	3620	570	180	359	2874	1140	285	71	90	3620	3620	1437	453	718
271671	2874	1810	570	359	285	718	71	45	22	22	3620	1437	905	359	570
198987	2281	1437	143	36	113	2281	285	36	18	57	2281	1810	570	226	180
323515	3620	2281	453	143	180	3620	3620	28	14	14	3620	2874	718	143	180
320888	2874	718	71	28	45	2874	570	180	57	45	1810	718	359	180	90
267765	2281	1810	570	285	285	905	90	18	14	14	2281	453	453	226	285
110443	3620	1437	226	113	180	2281	570	71	36	57	3620	3620	905	359	453
401522	2281	453	14	14	18	3620	453	45	14	45	2874	453	453	285	453
380501	1437	570	45	18	57	2874	718	90	113	71	1437	453	180	45	71
176972	3620	1810	453	180	285	570	180	14	14	14	3620	3620	285	143	180
336759	359	359	71	18	57	570	359	226	57	45	718	453	180	57	180
482490	905	45	18	14	14	453	45	14	14	14	3620	3620	2281	2281	718
477571	2874	905	180	71	180	1140	453	71	22	22	1810	2874	359	718	285
192130	905	1140	143	45	143	718	905	285	57	226	718	718	453	180	180
985	453	453	71	22	90	718	1140	113	45	143	1810	2281	905	180	453
394951	570	1140	90	22	90	718	718	453	71	113	570	905	113	143	143
346858	1810	1140	90	36	113	359	718	453	90	36	905	905	180	226	113
318775	905	1810	570	226	180	45	18	14	14	14	285	570	285	226	143
421648	2281	2281	113	28	22	3620	3620	359	143	90	1810	905	143	113	143
323845	718	285	90	22	22	1437	905	359	143	285	1437	2281	1810	905	905
379131	905	570	45	22	28	1810	905	143	57	45	3620	905	285	90	71
267871	905	180	14	14	18	718	90	113	36	45	570	113	113	36	71
267367	453	570	14	14	14	718	359	57	36	36	905	1437	453	180	453
230397	1810	126	22	14	28	2281	113	36	22	22	718	2281	1140	570	570
494837	113	71	14	14	14	359	180	22	18	18	3620	2281	226	226	226
**GMT**	**1488**	**803**	**122**	**56**	**81**	**1191**	**430**	**72**	**32**	**41**	**1958**	**1454**	**328**	**203**	**208**
**95% CI of GMT**	**1168-1896**	**592-1088**	**86-173**	**41-77**	**61-108**	**917-1545**	**310-598**	**54-96**	**26-38**	**34-51**	**1645-2330**	**1159-1824**	**253-426**	**159-259**	**167-259**
**% seroconverted (n)**	**100% (59/59)**	**100% (59/59)**	**92% (54/59)**	**83% (49/59)**	**92% (54/59)**	**100% (59/59)**	**100% (59/59)**	**86% (51/59)**	**70% (41/59)**	**80% (47/59)**	**100% (59/59)**	**100% (59/59)**	**100% (59/59)**	**100% (59/59)**	**100% (59/59)**

Supplemental Table S4. p values calculated from Wilcoxon matched-pairs signed-rank test for group comparison of NT_{50} geometric mean titers (GMTs) at each time-point from Figure 2B-E. Values >0.05 (not statistically significant) are highlighted in red.

Figure	Time-point	1 month (median 36 days) after 4th dose with ChAdOx1-S (Oxford/AstraZeneca)					4 months (median 126 days) after 4th dose with ChAdOx1-S (Oxford/AstraZeneca)					3 months (median 91 days) after Comirnaty Bivalent Original/Omicron BA.4/BA.5 (Pfizer-BioNTech)				
	**Variants**	**D614G**	**BA.4**	**XBB.1.5**	**XBB.1.16**	**EG.5.1**	**D614G**	**BA.4**	**XBB.1.5**	**XBB.1.16**	**EG.5.1**	**D614G**	**BA.4**	**XBB.1.5**	**XBB.1.16**	**EG.5.1**
2B-D	**D614G**	-	<0.0001	<0.0001	<0.0001	<0.0001	-	<0.0001	<0.0001	<0.0001	<0.0001	-	0.0209	<0.0001	<0.0001	<0.0001
	**BA.4**	<0.0001	-	<0.0001	<0.0001	<0.0001	<0.0001	-	<0.0001	<0.0001	<0.0001	0.0209	-	<0.0001	<0.0001	<0.0001
	**XBB.1.5**	<0.0001	<0.0001	-	<0.0001	<0.0001	<0.0001	<0.0001	-	<0.0001	<0.0001	<0.0001	<0.0001	-	<0.0001	<0.0001
	**XBB.1.16**	<0.0001	<0.0001	<0.0001	-	0.0017	<0.0001	<0.0001	<0.0001	-	0.0011	<0.0001	<0.0001	<0.0001	-	**0.7282**
	**EG.5.1**	<0.0001	<0.0001	<0.0001	0.0017	-	<0.0001	<0.0001	<0.0001	0.0011	-	<0.0001	<0.0001	<0.0001	**0.7282**	-
2E	**1mo post-4th dose**	-	-	-	-	-	**0.0967**	0.0064	**0.0555**	0.0075	0.0006	**0.3126**	0.0029	0.0001	<0.0001	<0.0001
	**4mo post-4th dose**	**0.0967**	0.0064	**0.0555**	0.0075	0.0006	-	-	-	-	-	0.0005	<0.0001	<0.0001	<0.0001	<0.0001
	**3mo post-5th dose**	**0.3126**	0.0029	0.0001	<0.0001	<0.0001	0.0005	<0.0001	<0.0001	<0.0001	<0.0001	-	-	-	-	-

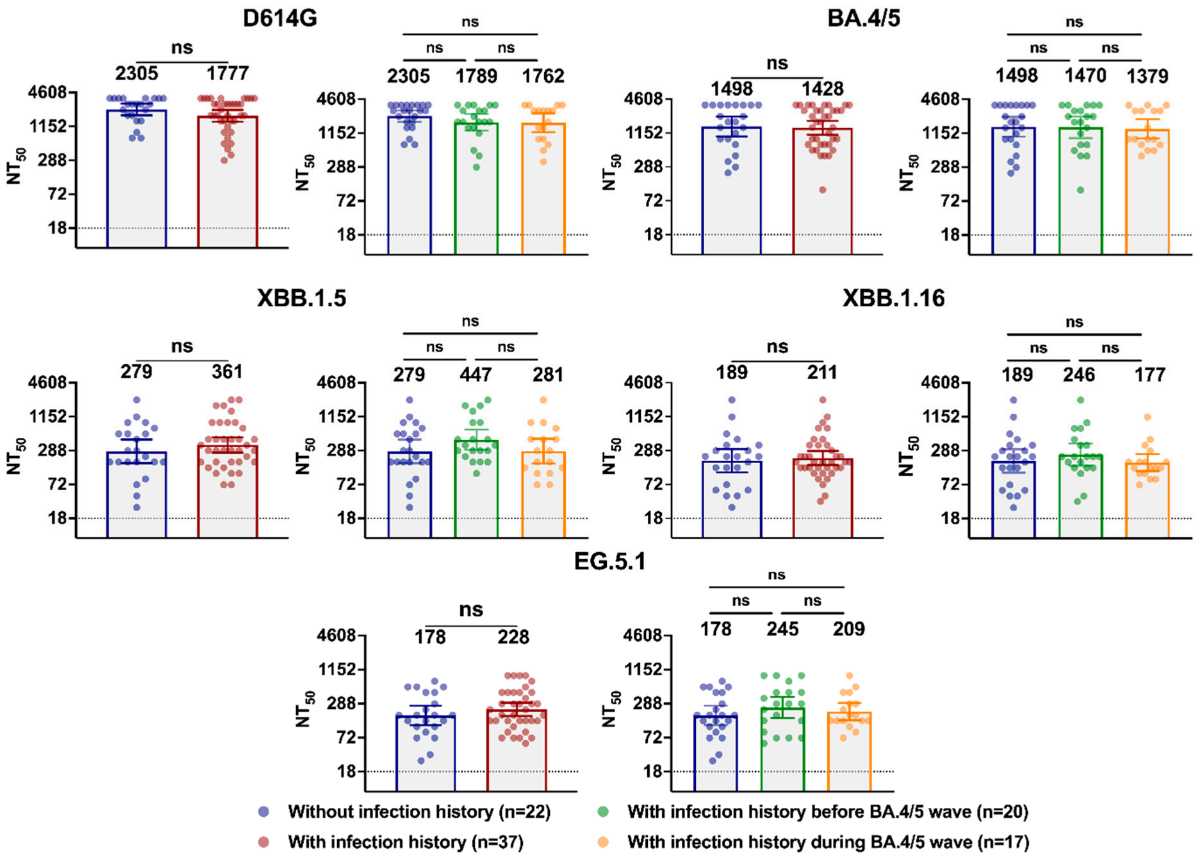

Supplemental Figure S1. Neutralization against Omicron sublineages BA.4/5, XBB.1.5, XBB.1.16 and EG.5.1 in individuals with or without history of infection by SARS-CoV-2. (A-B) NT_{50} values of 22 and 37 human sera without and with infection history, respectively, against Omicron BA.4/5, XBB.1.5, XBB.1.16 and EG.5.1, collected after **(A)** 1 month (median 36 days) post 4th dose with parental ChAdOx1-S vaccine, and **(B)** 3 months (median 92 days) post 5th dose with bivalent mRNA vaccine**.** Geometric mean titers (GMTs) are noted above the respective groups. Colors designate infection history, data points represent individual subjects, bars represents GMT with 95% confidence intervals (CI) and dotted lines represent the lower limit of detection of NT_{50} (LOD=18). Groups were compared by non-parametric Mann-Whitney test (ns = not significant, * = $p<0.05$). Panels (A-B) were generated using GraphPad Prism v10.0 software.

Supplemental Table S5. Geometric mean titers (GMTs) with 95% CI of neutralizing titers against SARS-CoV-2 ancestral strain (D614G) and Omicron sublineages BA.4/5, XBB.1.5, XBB.1.16 and EG.5.1 at 1 and 4 months post 4th dose with ChAdOx1-S (Oxford/AstraZeneca) and 3 months post 5th dose with Comirnaty Bivalent Original/Omicron BA.4/BA.5 (Pfizer-BioNTech). Individual NT_{50} values is the geometric mean (GMT) of triplicate CPE-VNT results (one independent experiment). Titers below the LOD (<20) were represented by half the LOD, 10 for plot purpose and statistical analysis. NT_{50}, 50% neutralization antibody titer; 95% CI, 95% confidence interval. p values calculated from nonparametric Mann-Whitney test (unpaired) for group comparison of individuals without and with infection history by SARS-CoV-2 of NT_{50} geometric mean titers (GMTs) at each time-point from Figure 3 and Supplemental Figure 1. p values <0.05 (statistically significant) are highlighted in red.

Time-point	Neutralazing antibodies titer (NT50) - GMT and 95% CI																			
	Without (n=22)					With (n=37)					With before BA.4/5 wave (n=20)					With during and/or after BA.4/5 wave (n=17)				
	D614G	BA.4	XBB 1.5	XBB 1.16	EG.5.1	D614G	BA.4	XBB 1.5	XBB 1.16	EG.5.1	D614G	BA.4	XBB 1.5	XBB 1.16	EG.5.1	D614G	BA.4	XBB 1.5	XBB 1.16	EG.5.1
1 month (median 36 days) after 4th dose with ChAdOx1-S (Oxford/AstraZeneca)	1452 (912-2309)	573 (296-1108)	100 (55-181)	46 (27-77)	61 (38-99)	1510 (1131-2016)	982 (730-1319)	138 (88-216)	63 (42-96)	96 (67-137)	1558 (1020-2378)	937 (591-1487)	184 (99-340)	81 (47-140)	116 (73-183)	1456 (942-2251)	1037 (694-1548)	98 (50-195)	47 (24-93)	77 (43-140)
4 months (median 126 days) after 4th dose with ChAdOx1-S (Oxford/AstraZeneca)	924 (584-1463)	279 (158-493)	53 (31-90)	26 (19-35)	31 (22-43)	1384 (1004-1907)	556 (373-828)	87 (63-120)	35 (27-46)	49 (38-64)	1388 (849-2269)	399 (222-716)	82 (51-131)	36 (25-51)	45 (31-65)	1379 (878-2165)	823 (486-1393)	93 (57-154)	35 (23-52)	55 (37-83)
3 months (median 91 days) after Comirnaty Bivalent Original/Omicron BA.4/BA.5 (Pfizer-BioNTech)	2305 (1817-2923)	1498 (999-2248)	279 (172-452)	189 (117-306)	178 (120-264)	1777 (1395-2263)	1428 (1074-1899)	361 (264-495)	211 (159-281)	228 (174-299)	1789 (1272-2517)	1470 (945-2288)	447 (297-675)	246 (156-387)	245 (160-377)	1762 (1202-2582)	1379 (932-2042)	281 (170-466)	177 (125-252)	209 (147-297)

Time-point	p values					p values					p values					p values				
	Without (n=22) vs With (n=37)					Without (n=22) vs With before BA.4/5 wave (n=20)					Without (n=22) vs With during and/or after BA.4/5 wave (n=17)					With before BA.4/5 wave (n=20) vs With during and/or after BA.4/5 wave (n=17)				
	D614G	BA.4	XBB 1.5	XBB 1.16	EG.5.1	D614G	BA.4	XBB 1.5	XBB 1.16	EG.5.1	D614G	BA.4	XBB 1.5	XBB 1.16	EG.5.1	D614G	BA.4	XBB 1.5	XBB 1.16	EG.5.1
1 month (median 36 days) after 4th dose with ChAdOx1-S (Oxford/AstraZeneca)	0.9717	0.2418	0.4104	0.3457	0.1783	0.9347	0.3499	0.1571	0.1133	0.0813	0.8705	0.2932	0.9274	0.9493	0.6372	0.8364	0.9819	0.1296	0.1293	0.2632
4 months (median 126 days) after 4th dose with ChAdOx1-S (Oxford/AstraZeneca)	0.0573	**0.0367**	0.1135	0.1485	**0.0346**	0.0675	0.2719	0.2605	0.17	0.1362	0.1711	**0.0109**	0.1137	0.2901	**0.0328**	0.9576	0.1135	0.9577	0.8244	0.4523
3 months (median 91 days) after Comirnaty Bivalent Original/Omicron BA.4/BA.5 (Pfizer-BioNTech)	0.1821	0.6621	0.5133	0.9284	0.3999	0.2374	0.9142	0.1869	0.5424	0.3183	0.283	0.5086	0.7728	0.6074	0.698	0.9697	0.7437	0.1532	0.1165	0.5487
